# Laboratory Evolution of GH11 Endoxylanase Through DNA Shuffling: Effects of Distal Residue Substitution on Catalytic Activity and Active Site Architecture

**DOI:** 10.3389/fbioe.2019.00350

**Published:** 2019-11-22

**Authors:** Ming-Qi Liu, Jia-Yi Li, Ashfaq Ur Rehman, Xin Xu, Zhu-Jun Gu, Ruo-Chen Wu

**Affiliations:** ^1^Key Laboratory of Marine Food Quality and Hazard Controlling Technology of Zhejiang Province, College of Life Sciences, China Jiliang University, Hangzhou, China; ^2^State Key Laboratory of Microbial Metabolism, School of Life Sciences and Biotechnology, Shanghai Jiao Tong University, Shanghai, China

**Keywords:** xylanase, DNA shuffling, catalytic activity, molecular dynamics simulations, non-covalent interactions (NCI), xylooligosaccharides

## Abstract

Endoxylanase with high specific activity, thermostability, and broad pH adaptability is in huge demand. The mutant library of GH11 endoxylanase was constructed via DNA shuffling by using the catalytic domain of *Bacillus amyloliquefaciens* xylanase A (BaxA) and *Thermomonospora fusca* TF xylanase A (TfxA) as parents. A total of 2,250 colonies were collected and 756 of them were sequenced. Three novel mutants (DS153: N29S, DS241: S31R and DS428: I51V) were identified and characterized in detail. For these mutants, three residues of BaxA were substituted by the corresponding one of TfxA_CD. The specific activity of DS153, DS241, and DS428 in the optimal condition was 4.54, 4.35, and 3.9 times compared with the recombinant BaxA (reBaxA), respectively. The optimum temperature of the three mutants was 50°C. The optimum pH for DS153, DS241, and DS428 was 6.0, 7.0, and 6.0, respectively. The catalytic efficiency of DS153, DS241, and DS428 enhanced as well, while their sensitivity to recombinant rice xylanase inhibitor (RIXI) was lower than that of reBaxA. Three mutants have identical hydrolytic function as reBaxA, which released xylobiose–xylopentaose from oat spelt, birchwood, and beechwood xylan. Furthermore, molecular dynamics simulations were performed on BaxA and three mutants to explore the precise impact of gain-of-function on xylanase activity. The tertiary structure of BaxA was not altered under the substitution of distal residues (N29S, S31R, and I51V); it induced slightly changes in active site architecture. The distal impact rescued the BaxA from native conformation (“closed state”) through weakening interactions between “gate” residues (R112, N35 in DS241 and DS428; W9, P116 in DS153) and active site residues (E78, E172, Y69, and Y80), favoring conformations with an “open state” and providing improved activity. The current findings would provide a better and more in-depth understanding of how distal single residue substitution improved the catalytic activity of xylanase at the atomic level.

## Introduction

Xylan is the most abundant hemicellulose in nature and is primarily composed of β-D-xylopyranosyl residues linked by β-1,4-glycosidic bonds. Endo-1,4-beta-xylanase (EC 3.2.1.8, referred hereinafter as xylanase) cleaves internal β-xylosidic glycosidic bonds in the main backbone of xylan, thereby releasing xylose, xylooligosaccharides (XOs), and small polymers from hemicellulose (Scheller and Ulvskov, 2010). The xylanases were classified into glycosyl hydrolase (GH) families 5, 8, 10, 11, 26, 30, and 43 according to the amino acid sequence similarities of catalytic domain (Henrissat and Davies, 1997). Generally, the GH10 xylanases typically have high molecular weight and own an (α/β)_8_ barrel fold, liking a “salad bowl,” while GH11 xylanases are characterized as low molecular mass and the “right-handed jellyroll” structure (Paës et al., 2012). Most of the GH11 xylanases lack xylosidase and cellulase activity and they were widely applied in food, feed, and paper pulp bleaching industries (Juturn and Wu, 2012).

As a biological catalyst, the catalytic activity and efficiency of xylanases are susceptible to be influenced by both external and internal factors. The most common external factors include temperature, pH, substrate, and inhibitors, while the internal factors include primary structure and crystal structure. Xylanases with improved properties (i.e., higher activity and stability) are urgently welcomed and needed for environmental, economic and industrial reasons (Pandey et al., 2016; Bala and Singh, 2019). It was reported that numerous strategies were applied to modify enzyme based on the structure-function relationship and included rational, semi-rational, and irrational design (directed evolution) (Farinas et al., 2001; Wong and Schwaneberg, 2003; Chica et al., 2005; Sarmiento et al., 2015, Cheng et al., 2015). Therefore, lots of researches confirmed that these strategies mentioned above could be adopted to enhance the catalytic activity and enzymatic properties of xylanase (Shibuya et al., 2000; Miyazaki et al., 2006; Stephens et al., 2007; Wang et al., 2013; Wahab et al., 2016; Prajapati et al., 2018; Damis et al., 2019).

*Thermomonospora fusca* TF xylanase A (TfxA) is one of the most thermostable GH11 xylanases. After being incubated at 75°C for 18 h, its residual activity was 96%. (Irwin et al., 1994). In 1989, the *tfxa* gene was recombinantly expressed in *Streptomyces lividans* and *Escherichia coli*; howbeit the activity of the recombinant enzyme was relative low (Ghangas et al., 1989). In comparison with the TfxA, the GH11 *Bacillus amyloliquefaciens* Xylanase A (BaxA) is mesophilic and less thermostable. The activity of TfxA is lower than that of plenty of GH11 xylanases such as BaxA and *Aspergillus niger* xylanase A (AnxA) (Xu et al., 2016). The sequence similarity index results revealed that the catalytic domain of TfxA is nearly 40% identical with that of BaxA. In our previous study, we have modified the *baxA* gene (KM624029), encoding the BaxA by using error-prone PCR (epPCR) (Xu et al., 2016). The mutant reBaxA50 (S138T, including signal peptide) with improved catalytic activity was screened and characterized.

DNA shuffling, as one of rapid and powerful techniques for directed evolution of enzymes, was discovered by Stemmer in 1994 (Stemmer, 1994a,b). In the study, the mutant library was constructed via DNA shuffling by using the catalytic domain (CD) of TfxA and BaxA as parents. Moreover, three mutants (DS153, DS241, and DS428) were identified, and the variation in the enzymatic properties between reBaxA and mutants were analyzed in detail. Additionally, we have explored the activation mechanism for the first time, induced by GOF-mutations using essential dynamics simulation approaches. Subsequently, non-covalent interactions (NCI) analysis was also carried out to deepen understanding of modeling distinctions at the atomic level. Exploring effects of distal residue substitution on active site architecture may assist in providing valuable information for better clarifying the mechanism of enhanced activity.

## Materials and Methods

### Materials

The recombinant pCold TF-*mbaxA* plasmid and pCold I-*tfxA_cd* plasmid were stored at −80°C in laboratory. The pCold TF vector, DNase I (RNase-free), T_4_ DNA ligase, PCR kit, and restriction endonuclease were purchased from Takara (Dalian, China). Oat spelt xylan (X0627), birchwood xylan (X0502), and beechwood xylan (X4252) were purchased from Sigma-Aldrich (Shanghai) Trading Co., Ltd. (Shanghai, China). Xylose (X) was obtained from Merck (Darmstadt, Germany). Standard xylooligosaccharides (X2 to X6) were procured from Megazyme (Wicklow, Ireland). Protein molecular weight markers and antibodies were supplied by Songon Biotechnology Co., Ltd. (Shanghai, China). High-affinity Ni-charged resin was provided by GenScript Biotechnology Co., Ltd. (Nanjing, China). The primers were synthesized at Shanghai Sunny Biotechnology Co., Ltd. (Shanghai, China).

### Construction of DNA Shuffling Mutant Library

The mutant library was generated as described by Shibuya with slight modifications (Shibuya et al., 2000). DNA fragments (*baxA* and *tfxA_cd*, about 600 bp) were amplified from pCold TF-*baxA* and pCold I-*tfxa_cd* plasmids with primers DNAScold1: 5′-TCGGTACTCTCGAAGGTTTCGAATTC−3′ and DNAScold2: 5′ -GTCCTTTTAAGCAGAGCTTACTATCTAGA-3′, which contained *Eco*RI and *Xba*I recognition sites (underlined), respectively. The PCR parameters were as followed: 95°C, 2 min; 32 cycles of (94°C, 50 s; 61°C, 50 s with a 0.2°C decline per cycle; and 72°C, 1 min); and 72°C, 10 min. The PCR products were purified and recovered. Two genes (*baxA*, 3 μg; *tfxA_cd*, 3 μg) were mixed and randomly digested by DNaseI (0.2 U) at 37°C for 15 min. The digested products were analyzed in 2.0% (w/v) agarose gel, and then DNA fragments with the molecular mass <100 bp were recovered and purified. The gel-recovered fragments were reassembled into full-length genes via primerless PCR under the following conditions: 94°C, 150 s; 40 cycles of (30 s at 94°C, 47.5 s at 61°C, 4 min at 72°C); 72°C, 10 min. The primerless PCR product was analyzed by electrophoresis and used as the template to amplify the single product of full-length (about 600 bp) by using the primers DNAScold1 and DNAScold2. Moreover, the full-length gene was purified and digested by *Eco*RI and *Xba*I. The digested full-length gene was inserted into pCold TF vector and then transformed into *E. coli* BL21 (DE3) competent cells. All of the transformed products were plated on lysogenic broth (LB) agar plates (ampicillin, 100 μg/mL) and cultured at 37°C for 12 h.

### Screening of the Mutant Library and Activity Assay

The colonies on LB agar plates (ampicillin, 100 μg/mL) were inoculated in 5 mL of LB medium (ampicillin, 100 μg/mL) at 37°C for 12 h and then 1 mL cultured cells were transferred into shake flask (50 mL of LB medium with 100 μg/mL ampicillin) continuously cultured at 37°C for 3 h. The shake flask was stored at 15°C for 30 min. In order to induce expression of xylanase, isopropyl-β-D-thiogalactopyranoside (IPTG) was added to the medium with a final concentration of 1.0 μM, and the cells were cultured under shaking (150 rpm) at 15°C for 24 h.

The fermentation supernatant was collected and used for enzyme activity assay in a 96-well plate as follows. First, 50 μL of 0.5% beechwood xylan and 20 μL of fermentation supernatant were co-incubated at 50°C for 5 min. Then, 50 μL of 3,5-dinitrosalicylic acid (DNS) was added to stop the reaction and placed in a boiling water bath for 5 min. After 100 μL of deionized water was added, absorbance was determined spectrophotometrically at 540 nm by using a multiscan spectrum microplate spectrophotometer (Xu et al., 2016). These colonies that showed xylanase activity were sequenced. Mutant DS153, DS241, and DS428 were characterized in details.

One unit of xylanase activity was defined as the amount that released 1 μmol of reducing sugar equivalents (D-xylose as the standard) per minute from beechwood xylan under optimal conditions (Miller, 1959; Miller et al., 1959). Protein concentration was measured by Bradford method with bovine serum albumin as the standard (Bradford, 1976). The Michaelis–Menten constant (*K*_*m*_), the maximum reaction rate (*V*_*max*_), and molar catalytic activity (*k*_cat_) of xylanase were determined from nonlinear regression fitting to the Michaelis-Menten equation by using beechwood xylan as substrate (1, 3, 5, 7.5, and 10 mg/mL). All assays were performed in triplicate to obtain the mean and standard deviation.

### SDS-PAGE and Western Blotting Analysis

The xylanase with high activity was purified using high-affinity Ni-charged resin from the culture supernatant. The samples were analyzed by sodium dodecyl sulfate-polyacrylamide gel electrophoresis (SDS-PAGE) with the stacking and separating gels consisting of 5% and 12% polyacrylamide, respectively (Laemmli, 1970). Proteins in the gel were stained with Coomassie brilliant blue R-250. Anti-His monoclonal and horseradish peroxidase-labeled goat anti-mouse IgG antibodies were used for Western blotting analysis (Huo et al., 2018).

### Optimum Temperature and Thermostability

The effect of temperature on the xylanase activity was determined from 30 to 90°C. For thermal stability assay, the xylanase was heat-treated from 30 to 90°C for 15 min and then placed in an ice-water bath for 5 min. Residual activity was determined under optimal conditions.

### Optimum pH and pH Stability

The effect of pH on xylanase activity was evaluated from pH 3.0 to 9.0 at 50°C. The highest activity was taken as 100%. For pH stability, xylanase was incubated in buffer solutions (pH 3.0–9.0) at 25°C for 1 h. Residual activity was measured under optimal conditions. For assays of pH stability and thermostability, the activity of the untreated xylanase under optimal conditions was taken as 100%.

### Inhibitory Activity of Recombinant Rice Xylanase Inhibitor on Xylanase

Rice xylanase inhibitor (RIXI) is one of the XIP-type xylanase-inhibiting proteins in rice, which involved in protecting rice from attacks by plant-pathogenic microorganisms and inhibiting most exogenous GH10 and 11 xylanases (Goesaert et al., 2005). The content of xylanase-inhibiting proteins in rice and wheat is relatively high, and theses inhibitors reduced the application performance of xylanase in the feed and food industries (Dornez et al., 2010). According to our previous study (Huo et al., 2018), we determined inhibitory activity of recombinant rice xylanase inhibitor (rePRIXI) on reBaxA, TfxA_CD, and three mutants (DS153, DS241, and DS428). Xylanases (0.4 U) were incubated with rePRIXI (0.2 mg) at 50°C for 40 min, and then the residual activity and the inhibitory rate were calculated.

### Hydrolysis of Xylans by Mutant Xylanase

Beechwood, birchwood, and oat spelt xylan (0.5 mL, 1.0%, w/v) were hydrolyzed by the three mutants (0.35 U) at 50°C for 15 min, respectively. The hydrolyates were analyzed via high-performance liquid chromatography (HPLC) with a Sugar-pak^TM^ I column and a Waters 2410 refractive index detector. Besides, standard xylose (X) and xylooligosaccharides (X2–X6) were also examined (Xu et al., 2016).

### All-Atom Molecular Dynamics Simulations

Appling the three-dimensional (3D) structure of GH11 *B. subtilis* 1A1 (PDB ID 1XXN) as the template, the structural coordinates of BaxA and mutants (DS153, DS241, and DS429) were obtained via homology modeling using the SWISS-MODEL server (www.swissmodel.expasy.org) (Waterhouse et al., 2018), followed by energy minimization using Discovery Studio 3.5. The sequence identity of BaxA with the template was 96.22%.

The stability of wild-type and mutant xylanases were evaluated through extensive molecular dynamics simulations using the Amber14 package with the *ff14SB* force field (Case et al., 2018; Rehman et al., 2019). The LEAP module was used to add hydrogen atoms to the crystal structures. Counterions (Na^+^ and Cl^−^) were added for maintenance of system neutrality. The system was first subjected to 10,000 steps of steepest descent energy minimization followed by 1,000 cycles of conjugate gradient minimization with bonds involving hydrogen constrained by SHAKE algorithm (Ryckaert et al., 1977), and then another 10,000 steps of steepest descent energy minimization followed by 5,000 cycles of conjugate gradient minimization with no constraint exerted. Then, the system was then gradually heated from 0 to 300 K through 25,000 iterations. After a 200ps-equilibrium in NPT ensemble, five 50-ns simulations (300 K, 1 atm) with different random seeds were conducted. The VDW interactions were cut off at 10 Å, and long-range electrostatic interactions were calculated with particle mesh Ewald (PME) method (Darden et al., 1993). The GPU supported pmemd code performed the production step of molecular dynamics simulation for each system (Gotz et al., 2012). Analyses of trajectories were performed using cpptraj in Ambertools14.

### Non-covalent Interactions (NCI) Analysis of Substituted Residue and Active Sites

In this study, the quantum theory of atoms in molecules (QTAIM) (Bader, 1990) analysis, independent gradient model (IGM) (Lefebvre et al., 2017) analysis, reduced density gradient (RDG) (Johnson et al., 2010) analysis, and molecular electrostatic potential (MESP) were carried out with the Multiwfn 3.6 program (Lu and Chen, 2012). Molecular plots were visualized by the VMD 1.9.3 program (Humphrey et al., 1996). In atoms in molecules (AIM) analysis, the points at where the gradient norm of function value is zero (except at infinity) were called critical points (CPs). The appearance of a (3, −1) type of critical point usually appeared on a bond path or between the atoms which had attractive interaction, hence called as bond critical point (BCP). According to the AIM theory, the electron density (ρ) and its Laplacian value (∇^2^ρ) were used to indicate the strength and nature, respectively.

RDG analysis is a prevalent method to reveal weak interlayer interactions. The definition of the RDG function is shown below; it is essentially a dimensionless form of electron density gradient norm function:

$$\begin{array}{l} \text{RDG}\left(\text{r}\right)=\frac{1}{{2\left(3\pi^{2}\right)}^{\frac{1}{3}}}\frac{\left|\nabla \rho \left(r\right)\right|}{{\rho \left(r\right)}^{\frac{4}{3}}} \end{array}$$

It is based on the RDG as a function of sign(λ_2_)ρ, where the sign(λ_2_) is the sign of the second eigenvalue of the electron density Hessian matrix. Negative values of sign(λ_2_)ρ indicate attractive interactions, whereas positive values suggest repulsive interaction. In the RDG analysis, the isosurfaces were colored according to the value of sign(λ_2_)ρ, where a BGR (blue-green-red) color scale was adopted. Blue color represents attractive or bonding interaction, green weak van der Waals interaction, and red repulsive interaction. All isosurfaces are colored according to a BGR scheme over the electron density range −0.05 a.u. < sign(λ_2_)ρ <0.05 a.u.

Lefebvre et al. proposed a handy way of visually studying interfragment and intrafragment interactions, named IGM. The method employs, in addition to ρ, quantities related to the first and second derivatives of the density. The IGM descriptor δg^inter^ is given by the difference between the first derivatives of the charge densities for the total system and the fragments:

$$\begin{array}{l} {\delta \text{g}\left(\text{r}\right)}^{inter}=|\nabla \rho^{\text{IGM},\text{inter}}|-|\nabla \rho | \end{array}$$

δg^inter^ > 0 indicates the presence of weak interactions and the magnitude of the descriptor at a point in space indicates the strength of the interaction (Contreras-García et al., 2011).

## Results and Discussion

### Construction and Screening of Mutant Library

**Figure 1:**
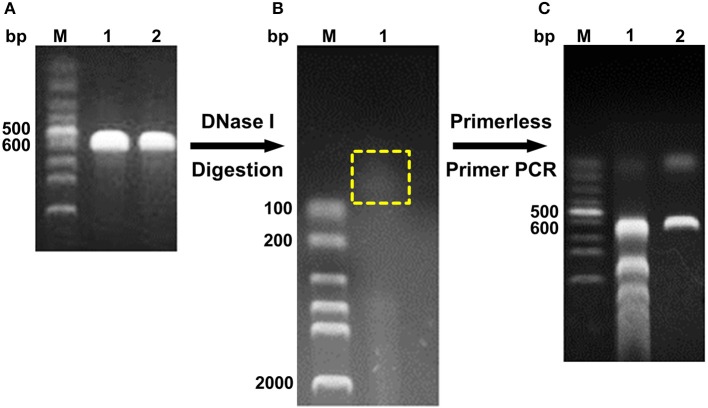
Schema of DNA shuffling. **(A)** PCR products of *baxa and tfxa_cd* gene. M, Lane 1, and 2 were DNA marker, *baxa, and tfxa_cd* gene, respectively. **(B)** Digestion of *baxa and tfxa_cd* gene by DNaseI. The digestion products in the yellow box were recovered. **(C)** Primerless/primer PCR product. Lane 1 was primerless PCR product by using the recovered digestion product as template. Lane 2 was PCR product by using primerless PCR product (lane 1) as template and DNAScold1, DNAScold2 as primers.

The digested products with a size of <100 bp were assembled through primerless PCR, and ~600 bp DNA was obtained (Figure 1). The following product of primerless PCR was further used as a template for amplification using both DNAScold1 and DNAScold2 as a primer. The PCR products were ligated into pCold TF vector and transformed into competent *E. coli* BL21 (DE3) cells. A total of 2,250 colonies were collected from the LB plates (ampicillin, 100 μg/mL). The mutant library was screened via 96-well plate method, and 756 colonies were sequenced and analyzed. The specific activities of DS153, DS241, and DS428 were 11.95, 11.45, and 10.26 U/mg, respectively. And they were 4.54, 4.35, and 3.9 times as high as that of reBaxA, respectively. The sequencing results for DS153, DS241, and DS428 revealed that the residues Asn29, Ser31, and Ile51 of BaxA were substituted by Ser38, Arg40, and Val59 of TfxA_CD correspondingly (Figure S1). There are not only many single site mutations in these sequenced mutants, but some fragment replacement mutations.

### SDS-PAGE, Western Blotting, and Kinetic Constant Analysis

**Figure 2:**
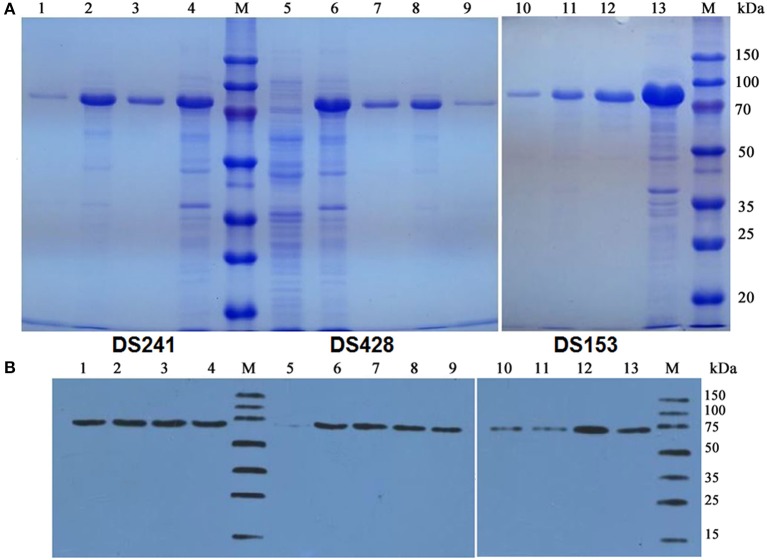
SDS-PAGE **(A)** and Western blot **(B)** analysis of mutant xylanases. M: standard protein marker; Lane 1, 9, and 10 were purified protein of DS241, DS428, and DS153 by Ni^2+^-chelating affinity chromatography, respectively; Lane 2, 8, and 11 were ultrasonication supernatant of DS241, DS428, and DS153 induced by IPTG, respectively; Lane 3, 7, and 12 were fermentation supernatant of mutant DS241, DS428, and DS153 induced by IPTG, respectively; Lane 4, 6, and 13 were cells of DS241, DS428, and DS153 induced by IPTG, respectively; Lane 5 was *E. coli* BL21 harboring the pCold TF vector induced by IPTG (control).

The pCold TF vector system provides a cold shock technology and a folding chaperone trigger factor for high-level expression of soluble recombinant proteins. The cold shock technology, which requires decreasing the culture temperature from 37 to 15°C during the induction period can suppress the expression of other cellular proteins and temporarily halt overall cell growth (Esposito and Chatterjee, 2006; Bjerga and Williamson, 2015). Xylanases were both secreted into the culture medium and remained in the cytoplasm of the three mutants after being induced by IPTG at 15°C. The results of SDS-PAGE and Western blotting unveiled a major band existed in the supernatant of the culture, and the proportion of the recombinant protein was high. The DS153, DSA241, and DS428 were further purified by Ni-affinity resin from culture supernatant with molecular weight approximately equal to 74.2 kDa (Figure 2).

Moreover, the reaction rates of the three mutants and reBaxA on beechwood xylan with different concentrations were plotted on Lineweaver–Burk graphs. Michaelis-Menten constant (*K*_*m*_) of DS428 was lower than that of reBaxA, whereas the *K*_*m*_ of DS153 and DS241 was slightly higher than that of reBaxA (Table 1). An increase in the *K*_*m*_ indicates the low affinity of xylanase for the substrate; it can be assumed that the alteration in affinity happens due to the substitutions of residues. The *k*_*cat*_/*K*_*m*_ of DS153, DSA241, and DS428 was higher than that of reBaxA, suggesting the enhanced catalytic efficiency.

**Table 1:** Kinetic constants of xylanase.

**Xylanase**	***V_**max**_* (μmol/mL.min)**	***K_**m**_* (mg/mL)**	***k_**cat**_* (1/s)**	***k_**cat**_/K_**m**_* (mL/mg.s)**
reBaxA	45.66 ± 1.912	16.05 ± 0.681	507.33 ± 9.212	31.61 ± 1.414
DS153	69.44 ± 2.281	17.24 ± 0.745	694.40 ± 12.212	40.28 ± 1.982
DS241	67.11 ± 2.320	16.32 ± 0.821	745.67 ± 15.212	45.69 ± 2.301
DS428	41.84 ± 2.224	12.05 ± 0.642	464.89 ± 13.212	38.58 ± 1.847

*Xylanase activities were determined at their optimum conditions described in section Materials and Methods. The concentration of beechwood xylan ranged from 1 to 10 mg/mL. Data was performed as the Mean ± SD (n = 3)*.

Shibuya et al. constructed the xylanase mutant library by using *S. lividans* xylanase B (SlxB-cat) and *T. fusca* xylanase A (TfxA-cat) as parents through DNA shuffling technique. Mutant Stx15 and Stx18 exhibited significant enhancement in thermostability at 70°C and their optimum temperature were equal to that of TfxA-cat. Moreover, their catalytic activities were improved in comparison with the parental xylanases. Sequencing results revealed that the 33 amino acids in N-terminus of Stx15 were from TfxA-cat and the other residues were from SlxB-cat, and the 62 amino acids in N-terminus of Stx18 were from TfxA-cat. Another mutant, Stx2 that had two residue substitutions (Q24P-Y170D) showed an enhancement in thermostability at 70°C (Shibuya et al., 2000).

### Optimum Temperature and Thermostability

**Figure 3:**
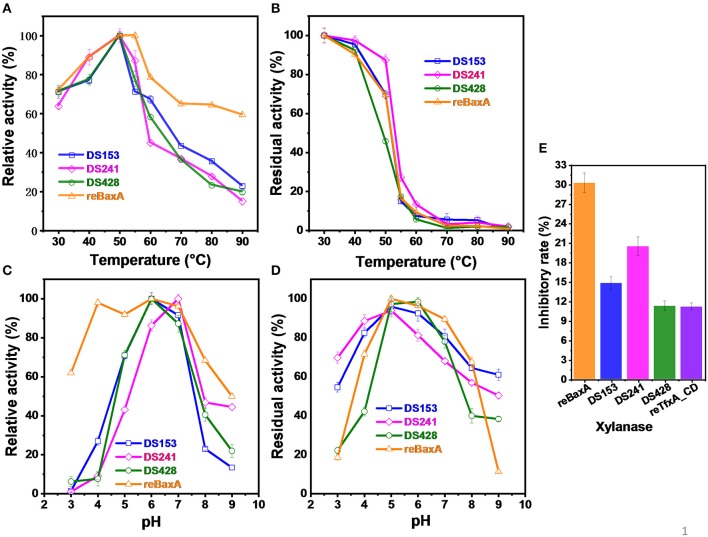
Enzymatic properties of reBaxA and three mutants. **(A)** Effect of temperature on activity of xylanases. 0.5% beechwood xylan was used as substrate. The highest activity was taken as 100% and the corresponding temperature was the optimum temperature. **(B)** Thermostability of xylanases. 0.5% beechwood xylan was used as substrate. The activity of xylanase under the optimum conditions was taken as 100% in assay of thermostability, and then the residual activity was assayed. **(C)** Effect of pH on activity of xylanases. 0.5% beechwood xylan in different pH buffer was used as substrate to explore effect of pH on activity. The highest activity was taken as 100% and the corresponding pH was the optimum pH. **(D)** pH stability. The activity of xylanase under the optimum conditions was taken as 100% in assay of pH stability, and then the residual activity was assayed. **(E)** Inhibitory activity of rePRIXI on xylanase. The rePRIXI (0.3 mg) was mixture with 5 types of xylanases (0.3 U) at 50°C for 40 min, respectively. And then the residual activity of xylanase was measured under their corresponding optimal condition. The inhibitory rate was obtained. Triplicate experiments were conducted for each assay.

The optimum temperatures of reBaxA and the three mutants (DS153, DS241, and DS428) were 55 and 50°C, respectively (Figure 3A). The reBaxA and three mutants showed high activity at low temperature (30–40°C), suggesting that these enzymes might be suitable for monogastric animal feed additives. The DS153 and DS241 were more thermostable compared with reBaxA. The residual activities for reBaxA, DS153, DS241, and DS428 were 69.42, 69.80, 87.41, and 45.78% respectively (Figure 3B) while treating them at temperature 50°C for 15 min. The strengthened thermostability was probably attributed to the mutation of S31R in DS241. The previous research found that the mutation from Ser to Arg and Asn to Ser were the most common mutations. So far as the consequences of these mutations, GH11 xylanases enhanced their catalytic activity and thermostability (Satyanarayana, 2013; Ayadi et al., 2015). Apart from that, the mutations (T11Y, N12H, N13D, F15Y, Y16F, S62T, S138T, S144C, N198D, A217V, and L49P) were discovered to be involved in conferring thermostability and catalytic activity on xylanases, including SoxB, SlxB, AnxA, AnxB, and BaxA (Zhang et al., 2010; Wang et al., 2012, 2015; Xu et al., 2016). Furthermore, incorporating the results of molecular dynamics simulations and visual inspection of protein modeling hierarchy revealed that the β-sheets of GH11 xylanases had high Arg contents and were more thermostable. The xylanase mutant MxylM4 with four Arg residues was obtained by substituting Ser and Thr with Arg residue. The *T*_1/2_ of the mutant MxylM4 at 80°C with the substrate increased from 130 to 150 min, and the *T*_*m*_ increased to 6°C (Satyanarayana, 2013).

### Optimum pH and pH Stability

The optimum pH of DS153 and DS428 was 6.0, which was the same as that of reBaxA. The optimum pH of DS241 was 7.0 (Figure 3C). GH11 xylanase catalyzes the hydrolysis step of xylan via the double-displacement mechanism, and two glutamic acid residues (E78 & E172 in BaxA) serve as a nucleophile and an acid/base catalyst. The optimum pH of GH11 xylanase was closely related to the residue (Asp or Asn) that formed hydrogen bonds with the acid/base catalyst residue (E172). Normally, the residue in xylanases with an optimum pH below 5.0 is Asp, whereas the residue in xylanases that are active under more alkaline conditions is Asn (Krengel and Dijkstra, 1996). The *A. niger* xylanase I and *T. fusca* TF xylanase A have optimum pH of 3.0 and 6.0, and their residues are D35 and N44, respectively. The *B. circulans* xylanase (BCX) and its mutant (N35D) have optimum pH of 5.7 and 4.6, respectively (Joshi et al., 2000). In BaxA, the N35 is the optimum pH-dependent residue, which is adjacent to the acid/base catalyst E172. The substitution of N29S in DS153 and S31R in DS241 did not affect the optimum pH; on the other hand, the substitution of I51V in DS428 changed its optimum pH from 6.0 to 7.0. Thus, it indicated that the DS153 and DS241 were more stable than reBaxA in the range from pH 3.0 to 9.0. The pH stability of DS428 at pH 3.0–9.0 was similar to that of reBaxA (Figure 3D).

### Effect of Recombinant RIXI on Xylanase Activity

The rePRIXI demonstrated different inhibitory activity on reBaxA, TfxA_CD and the three mutants. After incubating with rePRIXI at temperature 50°C for 40 min, the inhibitory rates of reBaxA, DS153, DS241, DS428, and TfxA_CD were 30.3, 14.81, 20.5, 11.32, and 11.2%, respectively (Figure 3E). Consequently, the substitution of N29S, S31R, and I51V relieved the xylanase inhibition by rePRIXI. The xylanases that possess a high tolerance toward inhibitors have relative merit in the hydrolysis of natural substrates, i.e., wheat bran, rice bran, and corncob. In the mutant library, there are some mutants associated with improved sensitivity toward rePRIXI. The rePRIXI inhibited 55.6%, 32.7%, 30.09%, and 29.5% of reBaxA50 (S138T, including signal peptide), DS454 (S100G in BaxA), DS214 (D20V-S64C-I114T in TfxA_CD) and DS526 (F14L-D20V-S64C-T144I-T185M in TfxA_CD), respectively (Liu et al., 2019).

The loop of RIXI (residues 144-153) inserted in the catalytic groove of GH11 xylanase and the residue Y147 and R148 directly interacted with the two catalytic residues, including E78 and E172 in BaxA. Our previous studies revealed that the inhibition of RIXI on BaxA belonged to competitive inhibition (Liu et al., 2019), and the molecular structural basis for the inhibition of BaxA by RIXI was similar to that of wheat XIP-I (Payan et al., 2004).

### Hydrolytic Products Released From Xylans by Xylanase

The hydrolytic products released from three xylans (beechwood, birchwood and oat spelt xylan) by the three mutants were determined by HPLC (Figure S2). The hydrolysates from beechwood, birchwood and oat spelt xylan by DS153, DS241, and DS428 were xylobiose (X2), xylotriose (X3), xylotetraose (X4), and xylopentaose (X5), which showed resemblance to those of reBaxA. The main product released from beechwood xylan and birchwood xylan by the three mutants was X3 (Table 2). Interestingly, the major product released by DS153, DS241, and DS428 from oat spelt xylan was X5. After hydrolysis at 50°C for 15 min, the concentrations of X3 from beechwood xylan by DS153, DS241, and DS428 were 1.729, 1.767, and 1.431 mg/mL, respectively (Table 2), which were higher than that of reBaxA (1.168 mg/mL). The concentrations of X5 from oat spelt xylan by DS153, DS241, and DS428 were 0.678, 1.018, and 0.459 mg/mL, respectively (Table 2). Among hydrolysates of oat spelt xylan, the concentration of X3 by DS241 was higher than that of DS153 and DS428. The main product from oat spelt xylan by reBaxA was X4, and the concentrations of X4 and X5 were 1.764 and 0.943 mg/mL, respectively.

**Table 2:** Concentration of hydrolyates of xylans.

**Xylan**	**Xylanase**	**Hydrolytic products concentration(mg/mL) and percentage content**			
		**X2**	**X3**	**X4**	**X5**
Birchwood	DS153	0.221 ± 0.0110	1.107 ± 0.0324	0.732 ± 0.0145	0.457 ± 0.0136
		(8.09%)	(45.19%)	(29.62%)	(17.10%)
	DS241	0.585 ± 0.0150	1.381 ± 0.0341	0.734 ± 0.0124	0.346 ± 0.0110
		(19.12%)	(45.87%)	(24.35%)	(10.66%)
	DS428	0.166 ± 0.0092	0.906 ± 0.0221	0.709 ± 0.0281	0.532 ± 0.0122
		(6.36%)	(40.65%)	(31.31%)	(21.68%)
Beechwood	DS153	0.404 ± 0.0184	1.729 ± 0.0381	0.988 ± 0.0254	0.538 ± 0.0098
		(10.80%)	(47.91%)	(27.48%)	(13.81%)
	DS241	0.834 ± 0.0114	1.767 ± 0.2580	0.838 ± 0.2410	0.360 ± 0.0211
		(22.15%)	(46.72%)	(22.28%)	(8.85%)
	DS428	0.303 ± 0.0412	1.431 ± 0.0241	0.887 ± 0.0140	0.518 ± 0.0127
		(9.30%)	(46.45%)	(28.74%)	(15.51%)
Oat spelt	DS153	0.094 ± 0.0015	0.577 ± 0.0089	0.483 ± 0.0045	0.678 ± 0.0052
		(3.97%)	(33.77%)	(27.16%)	(35.10%)
	DS241	0.494 ± 0.0042	1.028 ± 0.0451	0.523 ± 0.0101	1.018 ± 0.0198
		(16.13%)	(33.56%)	(17.07%)	(33.24%)
	DS428	0.106 ± 0.0012	0.404 ± 0.0071	0.419 ± 0.0052	0.459 ± 0.0060
		(7.64%)	(29.10%)	(30.19%)	(33.07%)

*The values are the average of the data obtained from the analyses; the error bars represent standard deviation. Data was represented as the Mean ± SD (n = 3)*.

The single substitution in the three mutants changed the major product of oat spelt and birchwood xylan, but did not change the kind of hydrolysates from the three xylans. The affinity of the three mutants toward beechwood and birchwood xylan was higher than that toward oat spelt xylan. Our previous study (Xu et al., 2016) revealed that reBaxA50 (S138T) obtained from epPCR mutant library has higher catalytic efficiency on beechwood and birchwood xylans than on oat spelt xylan.

XOs are used as functional additives in food and feed industries (Achary and Prapulla, 2011). X2–X5 are the key functional components of XOs. An *in-vivo* test on male Wistar rats has revealed that dietary XOs had positive influence on the development of aberrant crypt focal formation and metabolic abnormalities, which were related to colon cancer (Aachary et al., 2015). In an *in-vivo* test on laying hens, dietary XOs supplementation considerably increased egg production, improved calcium digestibility and eggshell quality, and decreased the levels of plasma glutamic-pyruvic transaminase, cholesterol and high-density lipoprotein (Li et al., 2017). The clinical observations in a pilot study have demonstrated that XOs could modify gut microbiota in healthy and prediabetic (Pre-DM) subjects. The XOs could remarkably decrease or reverse the increase in the abundance of *Howardella, Enterorhabdus*, and *Slackia* in healthy and Pre-DM subjects. In the Pre-DM group, the XOs reduced the two hrs-insulin levels in the oral glucose tolerance test but did not affect body composition, serum glucose, and satiety hormones (Yang et al., 2015). For this reason, the XOs can effectively maintain the health of human and animals, respectively.

### Structural Dynamics Features and Analysis of Underling Mechanisms

**Figure 4:**
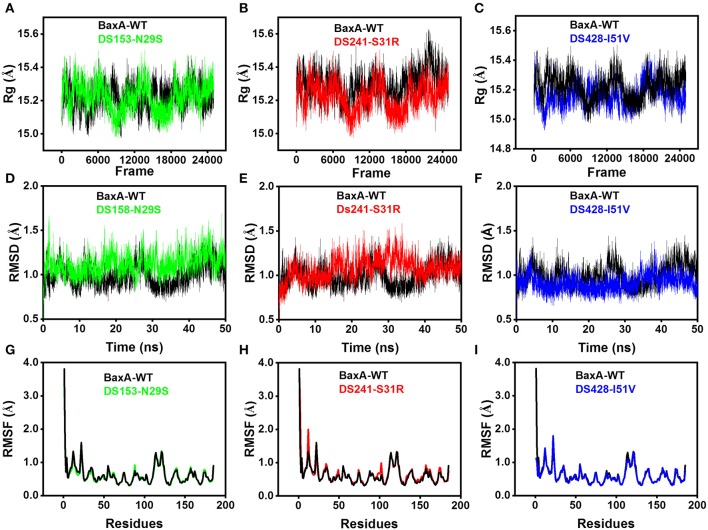
Superposed plots for radius of gyration (Rg), RMSD and RMSF of BaxA and three mutants. **(A–C)**, Superposed Rg graph. **(D–F)** Superposed RMSD graph. **(G–I)** Superposed RMSF graph. BaxA-WT (black), DS153-N29S (green), DS241-S31R (red), and DS428-I51V (blue).

Molecular dynamics (MD) simulations have been widely used to study the conformational changes of protein at the atomic level. The conformational features of all the systems were analyzed using MD trajectories along with the incorporation of various statistical parameters (Lobanov et al., 2008). The compactness of protein was measured by the radius of gyration (Rg). If a protein is stably folded, it will likely maintain a relatively steady value of Rg, whereas it will change over time for unfolded proteins (Rogerson and Arteca, 2011). The Rg results for wild-type and three mutants showed a slight difference in their steadiness (Figures 4A–C), with similar average Rg value (around 15.2 Å) during the simulation time, suggesting relatively stable conformation.

Root-mean-square deviation (RMSD) analysis gave insights into its structural conformation during the simulations, providing an indication of the stability of the protein and whether the simulation has equilibrated. The average backbone RMSD for WT and mutants were between 0.6 and 1.6 Å and remained stable through the entire MD simulations period (Figures 4D–F). Stable RMSD of the protein till the end of the simulation, suggested that the simulations were suitable for further rigorous analysis. In addition, the root-mean-square fluctuations (RMSF) were analyzed to figure out the impact of individual residues in both WT and MT xylanases (Figures 4G–I). There is slight residue fluctuation in regions adjacent to the mutation site. The increased variations in fluctuation in mutants indicated that point substitution (S31R, N29S, and I51V) involved in slight local structure change of xylanase. The three mutants and BaxA shared similar 3D model of GH11 xylanase, which resembled a β-jelly-roll fold or a partially closed right hand (Figure S3).

In DS153, DS241, and DS428, the conformations of local residues showed variation, i.e., in the wild type, van der Waals interaction was observed between S31 and N32, but in DS241, S31 mutating to R31 vanished the interaction between R31 and N32, instead introduced the interaction between the guanidinium group of R31 and N29 (Figures S4A–C). We also plotted electrostatic potential colored van der Waals surface map and penetration graph of van der Waals surfaces. It could be clearly seen that due to formation of non-covalent interaction, there was inter-penetration between the van der Waals surfaces of the R31 and N29 (Figure S4D). The NCI caused the van der Waals surfaces of the molecules to penetrate each other. Between R31 and N29, non-bonded radius of NH2_R31_, OD1_N29_ and the mutual penetration distance were 1.833, 1.705, and 0.453 Å, respectively. This was a non-trivial value, indicating the van der Waals force is obvious. However, in the I51V and N29S, there was no significant change in the environment around the mutated amino acid residues.

From the angle of protein structure, it was noticed that a residue substitution might have a dramatic effect on protein structure and function, especially in active site or cryptic site which occupied near to substrate-binding site (Worth et al., 2009). Distal single or several amino acid mutation/mutations may induce the change in molecular structure of enzyme, and the consequences could lead to opening or closing of the active site cleft (Shibuya et al., 2000; Zeymer and Hilvert, 2018). Hence, probing the impact of the substitution of distal residue on the active site architecture can provide valuable information for better understanding the altered protein structure and the function phenomenon.

**Figure 5:**
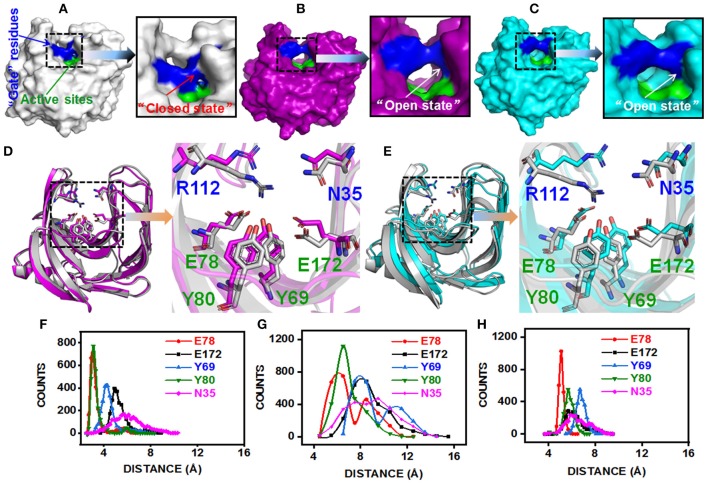
Post-MD modeling presentation and key residues distance analysis of BaxA, DS241, and DS428. Surface representation of BaxA-WT, DS241, and DS428: **(A)** BaxA-WT (gray) in “closed state.” **(B)** DS241-S31R (purple) in “open state.” **(C)** DS428-I51V (cyan) in “open state.” **(D)** Key residues (R112, N35, E78, E172, Y69, and Y80) of BaxA and DS241. **(E)** Key residues (R112, N35, E78, E172, Y69, and Y80) of BaxA and DS428. Key residues were shown as a stick model. R112 and N35 belonged to the “gate” residues. E78, Y69, Y80, and E172 belonged to the active sites. The plots (**F**, BaxA; **G**, DS241; **H**, DS428) showed the dramatic change in distance along MD simulations time, and the frequency distribution of distances between residue R112 and other key residues (E78, E172, Y69, Y80, and N35). Protein structures were generated and viewed by PyMOL 2.2.0 (DeLano, 2002) and Chimera 1.13 (Pettersen et al., 2004).

The substitution of N29S (DS153) and S31R (DS241) occurred in A3 β-strand which was in the “finger” structural region, while the substitution of I51V (DS428) occupied in A5 β-strand which was on the “palm” structural region (Figure S3). However, they could affect the architecture of the catalytic cleft based on the atomistic pair-wise distance analysis (Figures 5A–E). The NH1 atom of R112, OH atom of E78, E172, Y69, and Y80 were chosen as the representative atom. To elucidate a clear picture of the dynamic behavior along MD trajectory, the distribution of the atomic distance between R112-NH1 and E78-OH, E172-OH, Y69-OH, Y80-OH in WT and MT (DS241 and DS428) was calculated (Figures 5F–H). As a consequence of post-MD extracted protein structures, it was observed that the distance between both groups remained steady (from 3.5Å to 5.5Å) in the BaxA, showing the native conformation (“closed state”) of wild-type enzyme. However, in S31R and I51V system, the interface distance was far (from 6 to 8 Å), in which the residue R112 and N35 were flipped open for favoring the protein conformation in an “open state” and ultimately lost the strong interface interactions.

The solvent-accessible surface area (SASA) analysis also revealed the same mechanism too. The SASA is a parameter which measures the fraction of the protein surface interacting with the solvent molecules and could be used for predicting the extent of the conformational change. The SASA was analyzed using the cppptraj module in Amber Tools implemented linear combination of pairwise overlaps (LCPO) algorithm to explore how the distal substitution concealed or increased the accessibility of the catalytic site. Compared with BaxA, R112 slightly moved away from catalytic residues in DS241 and DS428, hence further boosting the SASA of mutants (Figure S5), indicating that once contacts of interface residues diminish or weaken, the SASA of the interface region will increase. Protein structure and dynamics may be beneficially changed by mutations that are distant from the active sites (Zeymer and Hilvert, 2018). These results delineate that the “open state” further promoted the catalytic efficiency to beechwood xylan.

### NCI Between “Gate” Residues and Active Site Residues

**Figure 6:**
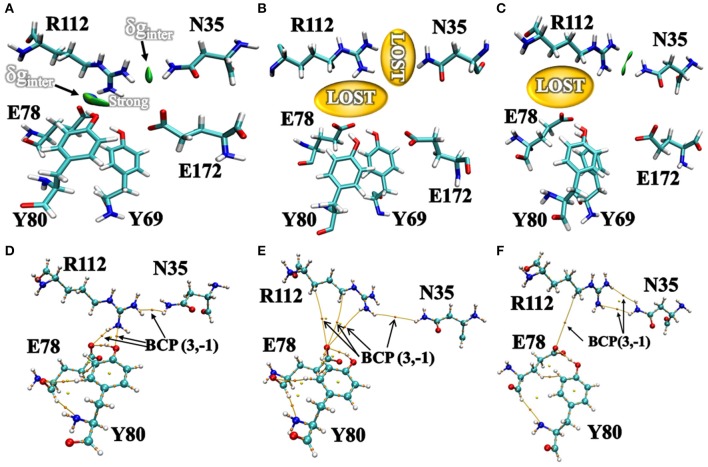
Intermolecular interactions between “gate” residues and active site residues. **(A)** BaxA; **(B)** DS241; **(C)** DS428. The blue represented a strong attraction, and red denoted a strong repulsion. All isosurfaces were colored according to a BGR (blue-green-red) scheme over the electron density range −0.05 a.u. < sign(λ_2_)ρ < 0.05 a.u. Bond critical points (BCPs, small orange spheres) using AIM analysis for different models **(D)** BaxA; **(E)** DS241; **(F)** DS428.

Additionally, we have analyzed the NCI to elucidate the interface residues interaction between “gate” residues (R112 and N35) and the active site architecture residues (E78, E172, Y69, and Y80). Compared to the popular NCI method, such as RDG, the inter-fragment interactions could be studied individually. Therefore, mutual interference was avoided by IGM method. The value of δg function in the interaction region directly reflected the strength of the interaction (Lefebvre et al., 2017). The ellipsoids were intermolecular interaction, the green and blue part generally represented the dispersion force and electrostatic force, respectively. It was intuitive that there was interaction between the “gate” residues and the active site architecture residues in BaxA. There was a vast green oval region in R112/E78 and R112/Y80 (Figure 6A). But in DS241 and DS428, single site substitution (S31R and I51V) induced a dramatically rotameric conformational change of “gate” residues, in which most of the residues completely lost the atomic interactions (R112/E78 and R112/Y80) as shown in Figures 6B,C, and there was not revealing area of a green oval. Additionally, it was intuitive that the residue pair R112/N35 was still involved in van der Waals interactions in DS428.

Topological analysis of the interface residues has been carried out to study the NCI among the residue pairs. Table 3 showed that there were topological properties at the bond critical points (BCPs) including the parameters of electron density (ρ), Laplacian of electron density (∇^2^ρ), kinetic energy density (G), and potential energy density (V) at the representative BCPs. Several BCPs among R112/E78, R112/Y80, R112/N35 can be observed in Figures 6D–F. In Table 3, it was noticed that the residue R112 and E78 attempted to form NCI in BaxA. The ρ value between residue R112-NH1 and Y80-OH was 0.7256 a.u., but in DS241 and DS428, the ρ and ∇^2^ρ-values were not in the range where weak interaction could be possible to form. Although the atoms of HH11, HD2, and HB2 in residue R112 and the OE1 in E78 formed a weak van der Waal's force in DS241, the value of ρ was minimal. In addition, in DS428, the HD3 atom of residue R112 formed van der Waals interaction with the OE1 of residues E78, where the value of ρ was 3.48E-06 a.u. It can be showed that this residue pair's interaction was still weaker, compared with the BCP intensity formed by residue R112 and E78 in BaxA. Therefore, the results of AIM further confirmed and suggested that the residue R112 in BaxA attempted to form an extensive interaction with residues in the catalytic cleft which did not exist in the DS241 and DS428.

**Table 3:** Comparison of the values of topological descriptors.

**Xylanase**	**Type**	**ρ(r) (a.u.)**	****∇^2^ρ**(r) (a.u.)**	**V(r) (a.u.)**	**G(r) (a.u.)**
BaxA	NH1_R112_-OH_Y80_	0.7256	0.2731	−0.5325	0.60765
	NH2_R112_-HD21_N35_	0.005235	0.02087	−0.002893	0.004056
	OE1_E78_-HH11_R112_	3.87E-06	4.70E-05	−1.42E-06	6.58E-06
DS241	OE1_E78_-HD2_R112_	3.75E-06	5.35E-05	−2.01E-06	7.70E-06
	OE1_E78_-HB2_R112_	−1.40E-06	3.50E-05	−1.40E-06	5.07E-06
	HH12_R112_-HD21_N35_	2.95E-05	1.62E-04	−9.63E-06	2.51E-05
	OE1_E78_-HD3_R112_	3.48E-06	4.85E-05	−2.00E-06	7.06E-06
DS428	OE1_E78_-HD3_R112_	3.48E-06	4.85E-05	−2.00E-06	7.06E-06
	NH1_R112_-HD21_N35_	1.48E-03	6.45E-03	−6.70E-04	1.14E-03
	HH22_R112_-HD22_N35_	1.32E-03	5.82E-03	−5.56E-04	1.01E-03

*Electron Density (ρ), Laplacian of the Electron Density (∇^2^ρ), Potential Energy Density [V(r)] obtained for the BCPs between “gate” residues and catalytic cleft sites residues in BaxA, DS241, and DS428*.

**Figure 7:**
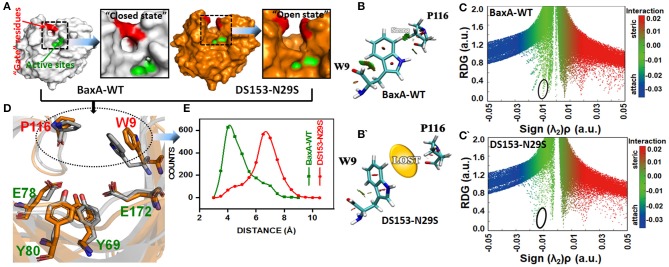
Post-MD modeling presentation and frequency distribution of distances between “gate” residues in DS153. **(A)** the cartoon representation to illustrate the variation in “gate” residues (W9 and P116) in BaxA and DS153. **(B,B′)** RDG isosurfaces for the WT and DS153 were individually plotted by using VMD 1.9.3. The green indicated van del Waals-type interactions. **(C,C′)** color-mapped scatter map was used to facilitate identification of correspondence between spikes and RDG isosurfaces using gnuplot program. **(D)** The stick models represented the location of crucial “gate” residues. W9 and P116 were substrate-binding subsites and flipped open allowing the substrate access the catalytic cleft. **(E)** The frequency distribution of distances between P116 and W9 in DS153 and BaxA.

To elucidate the impact of the substitution of N29S on the interaction of “gate” residues (W9 and P116), distribution of the residue pair's distance was calculated. For BaxA, W9 and P116 were in proximity to each other (close ensemble), remaining steady around 4 Å. However, the distance tremendously increased to ~7 Å and dramatically reduced the NCI (open ensemble) in DS153 (Figures 7A,D,E). Change in SASA (Figure S5) also demonstrated substitution of N29S could inevitably cause the hydrophobic residues (W9 and P116) to be exposed to the aqueous surrounding. The 3D RDG isosurfaces with BGR color scales representing sign(λ_2_)ρ values were showed in Figures 7B,B′. It could be seen that the intercommunications between residue W9 and P116 were prevailed by van der Waals interaction (represented in green) in BaxA. However, in DS153, the interaction was completely lost. In order to quantitatively understand the interlayer interactions, the dependence of RDG on sign(λ_2_)ρ was also calculated with a color-scale from −0.03 to 0.02 a.u. as shown in Figures 7C,C′. Accordingly, it could be seen that many RDG spikes in scatter graph, were shown as a function of sign(λ_2_)ρ from negative to positive values, as well as the spikes corresponding to H-bonding interactions, van der Waals interactions and steric effects. Compared with BaxA, DS153 mutant lost the spike corresponding to W9-P116 (Figures 7C,C′).

Possibly, GH11 xylanases had six sugar-binding subsites from −3 through +3 and preferentially bound more extended chain substrate (Brusa et al., 2018). The positive and negative numbers represented reducing and non-reducing end of the substrate, and the cleavage site was between the subsite −1 and +1, respectively (Wu et al., 2018). The residue R112 and N35 are the essential residues which attempt to form the −2 subsite (Brusa et al., 2018). And the N35 is the optimum pH-dependent residue of many GH11 xylanase. For the *B. circulans* xylanase (BCX), mutation of N35D shifted its pH optimum from 5.7 to 4.6, which further associated with increased activity (Joshi et al., 2000). The W9 and P116 were residues at subsite −2 and −1 of BaxA, respectively. The Crystal structure of BCX-xylotetraose complex revealed that the C5 of xylose residue 1 (xyl-1) bound with the CE2 and NE1 of W9 through hydrogen bond. The O3 atom of xylose residue 2 (xyl-2) interacted with NH2 atom of R112 and O of P116 with short hydrogen bond. Therefore, according to observations mentioned above, it could be delineated that the residue R112 played an essential role in the BCX, while mutation of N112 caused gain-of-loss (GOL) catalytic activity at a level of 65%, respectively (Wakarchuk et al., 1994).

## Data Availability Statement

All data generated or analyzed during this study are included in this published article and its Additional files.

## Author Contributions

M-QL designed the xylan hydrolysis experiments and supervised the study. J-YL is a collaborator in the MD simulations and structure analysis. AR helped in writing the manuscript and refining the graphs. XX performed the mutant library and enzymatic properties. Z-JG and R-CW performed the purification and inhibitory activity assay.

### Conflict of Interest

The authors declare that the research was conducted in the absence of any commercial or financial relationships that could be construed as a potential conflict of interest.

## Abbreviations

GH: glycosyl hydrolase
TfxA: *Thermomonospora fusca* TF xylanase A
TfxA_CD: catalytic domain of TfxA
*tfxa-cd*: gene encoding TfxA_CD
BaxA: *Bacillus amyloliquefaciens* xylanase A
*baxa*: gene encoding BaxA
AnxA: *Aspergillus niger* xylanase A
IPTG: isopropyl-β-D-thiogalactopyranoside
SDS-PAGE: sodium dodecyl sulfate-polyacrylamide gel electrophoresis
HPLC: high-performance liquid chromatography
XOs: xylooligosaccharides
X: xylose
X2: xylobiose
X3: xylotriose
X4: xylotetraose
X5: xylopentaose
X6: xylohexaose
*K*_*m*_: Michaelis–Menten constants
*V*_*max*_: maximal activity
*k*_cat_: molar catalytic activity
RIXI: rice xylanase inhibitor
MD: molecular dynamics
RMSD: root-mean-square deviation
RMSF: root-mean-square fluctuations
WT: wild-type
MT: mutant type
NCI: non-covalent interactions
Rg: radius of gyration
AIM: atoms in molecules
QTAIM: quantum theory of atoms in molecules
BCPs: bond critical points
IGM: independent gradient model
RDG: reduced density gradient
MESP: molecular electrostatic potential
SASA: solvent-accessible surface area
GOL: gain-of-loss.
