# Supplementary material for: Laboratory Evolution of GH11 Endoxylanase Through DNA Shuffling: Effects of Distal Residue Substitution on Catalytic Activity and Active Site Architecture
Source: Front Bioeng Biotechnol. 2019 Nov 22;7:350. doi: 10.3389/fbioe.2019.00350 (PMC6883096; doi:10.3389/fbioe.2019.00350)
Supplement: Supplementary file 1 [file Data_Sheet_1.docx]

**Supplementary material**

**Figure S1. Multiple sequence alignment of BaxA, TfxA_CD with three mutants.** Amino acid sequences were shown by single letters. The Multiple sequence alignment was assayed by the CLUSTAL Omega (https://www.ebi.ac.uk/Tools/msa/clustalo/) (Madeira et al., 2019). The “*” means the identical and fully conserved amino acid residue; “:” means conservation within a strong group of amino acid residue; “.” means conservation within weaker groups of amino acid residue. Three point mutations were unlined in red (bold), and the two catalytic sites of xylanase were marked in green (bold).

**Figure S2 Hydrolyates released from xylans by xylanase.** Hydrolysis products released from different xylans by mutants were detected by HPLC. **(A)**, Beechwood xylan. **(B)**, Birchwood xylan. **(C)**, oat spelt xylan. **(D)**, Standard X-X6. The position of xylose, xylobiose, xylotriose, xylotetraose, xylopentaose, and xylohexaose (X-X6) are shown.

**Figure S3 Modeling structure of BaxA.** β-jelly-roll fold of BaxA was shown as cartoon. BaxA composed of two anti-parallel β-sheets and a α-helix. The β-strands were marked as A series (A2, A3, A4, A5, A6) and B series (B2, B3, B4, B5, B6, B7, B8, B9). Stick representation included the catalytic residues (E78, E172), and the three mutant sites (N29, S31, and I51). The F, P, and T were the “fingers”, “palm”, and “thumb” domain of BaxA, respectively.

**Figure S4 Change in weak interaction around the S31R. (A),** Hydrogen bond existed between T15 and S31 in BaxA (in yellow) and in DS241 (in slate). S31 interacted with N32 via hydrogen bond in BaxA, which did not exist in DS241 between R31and N32. In addition, R31 interacted with N29 via van der Waals interaction in DS241, which did not exist in BaxA between S31and N29. **(B),** Color-filled RDG map for BaxA. The two hydrogen bonds between T15 and S31 should be strong, since corresponding RDG isosurfaces have blue color. **(C),** Color-filled RDG map for S31R. Green zone in an isosurface implied that the corresponding interaction is weak and may be regarded as van der Waals interaction. **(D)**, Plotting electrostatic potential colored van der Waals surface map and penetration graph of van der Waals surfaces.

**Figure S5 Frequency distribution of the SASA of BaxA and three mutants.** Results in solvent accessibility surface area (SASA) supported that substitution of S31R, N29S, and I51V changed the conformation of “Gate” residues and active site residues, which consequently enhanced the SASA.

**Figure S1**

**
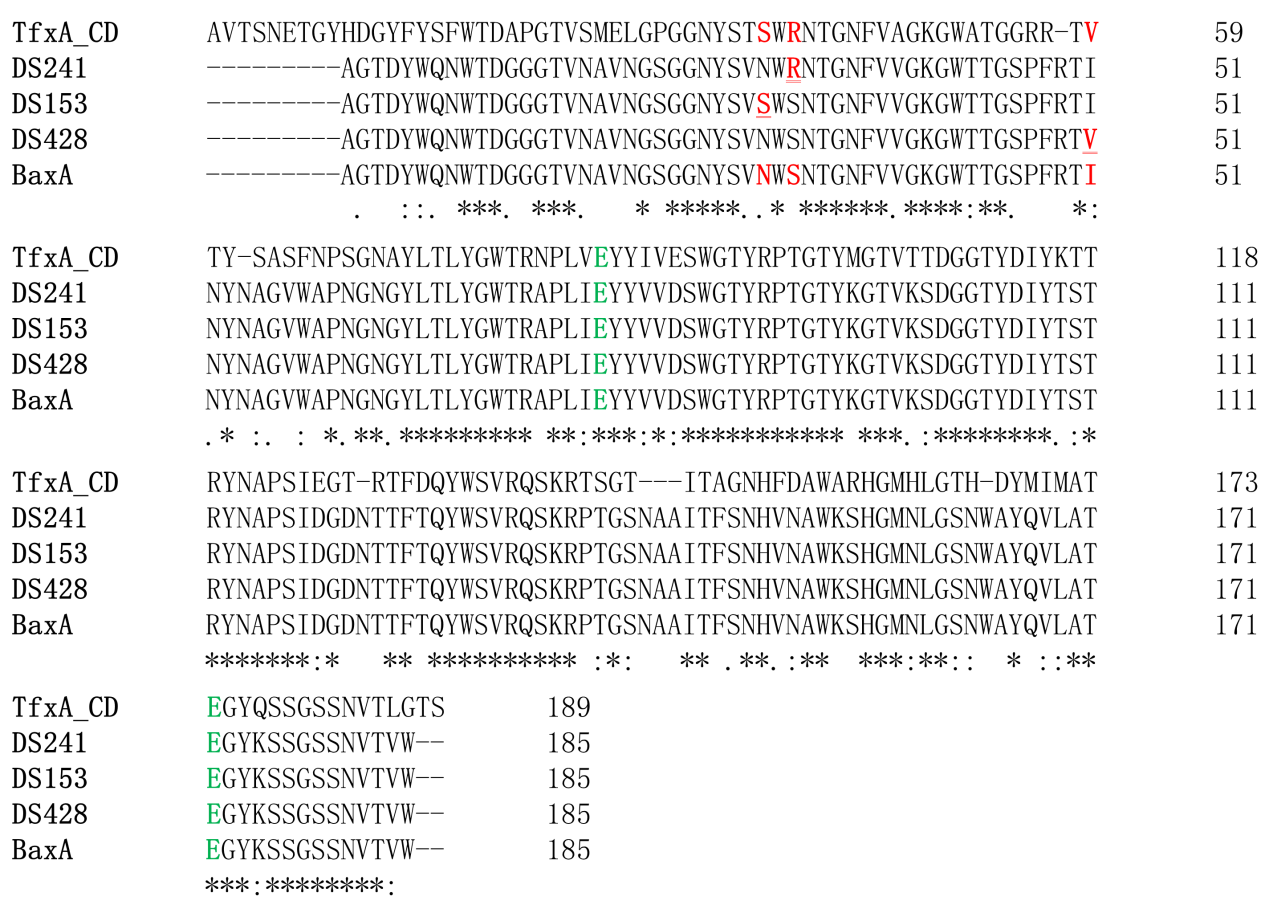
**

**Figure S2**


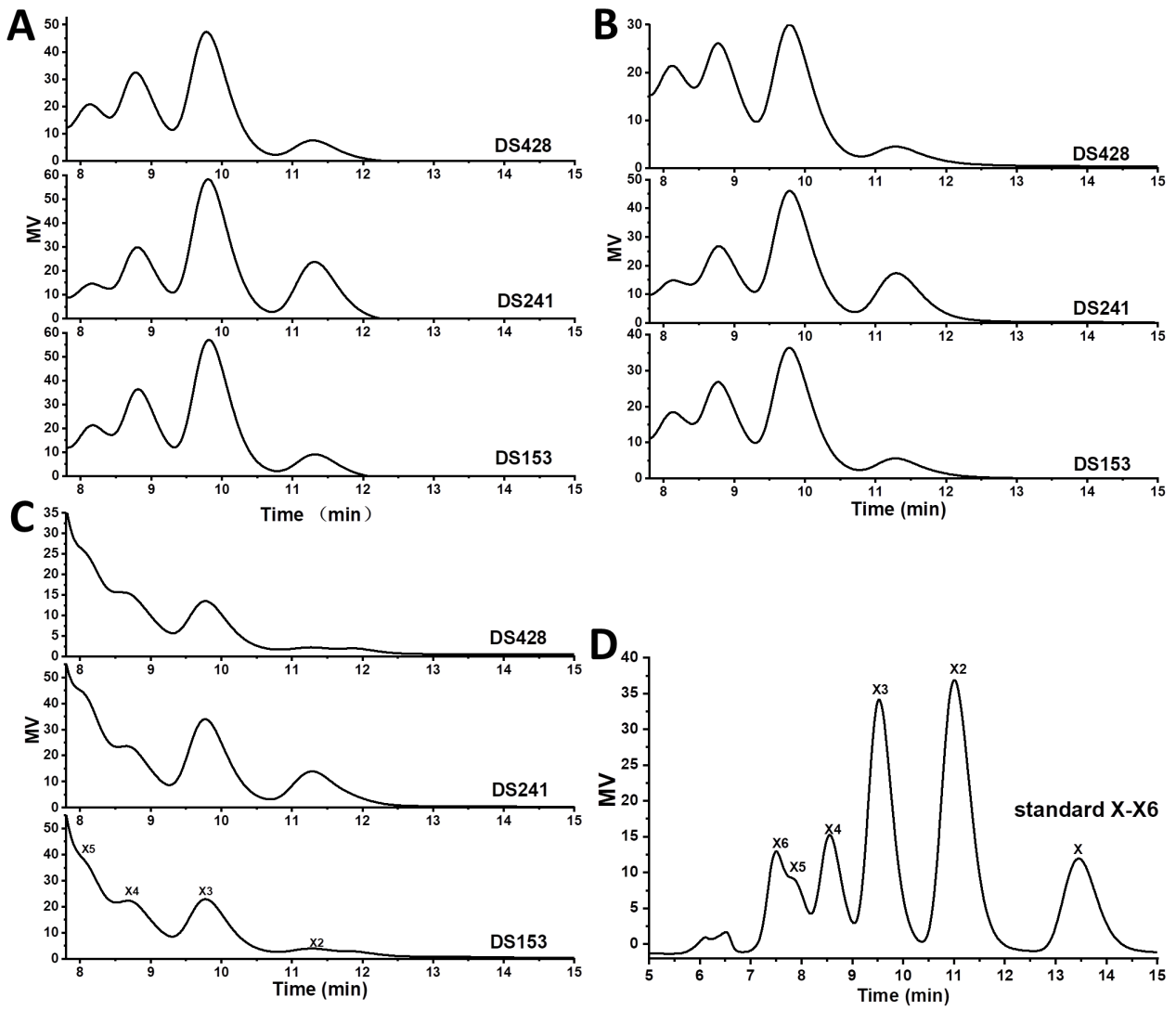


**Figure S3**

**
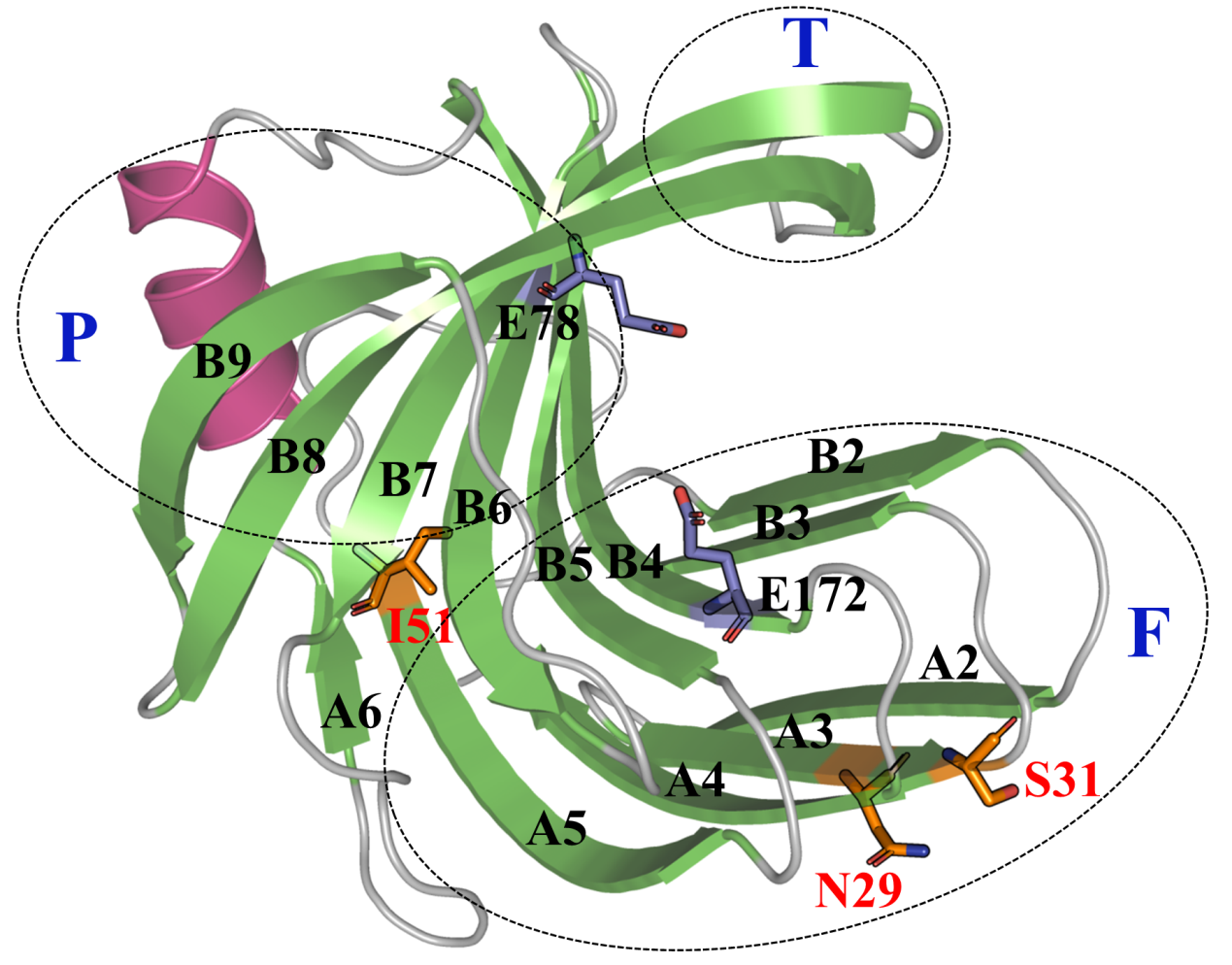
**

**Figure S4**

**
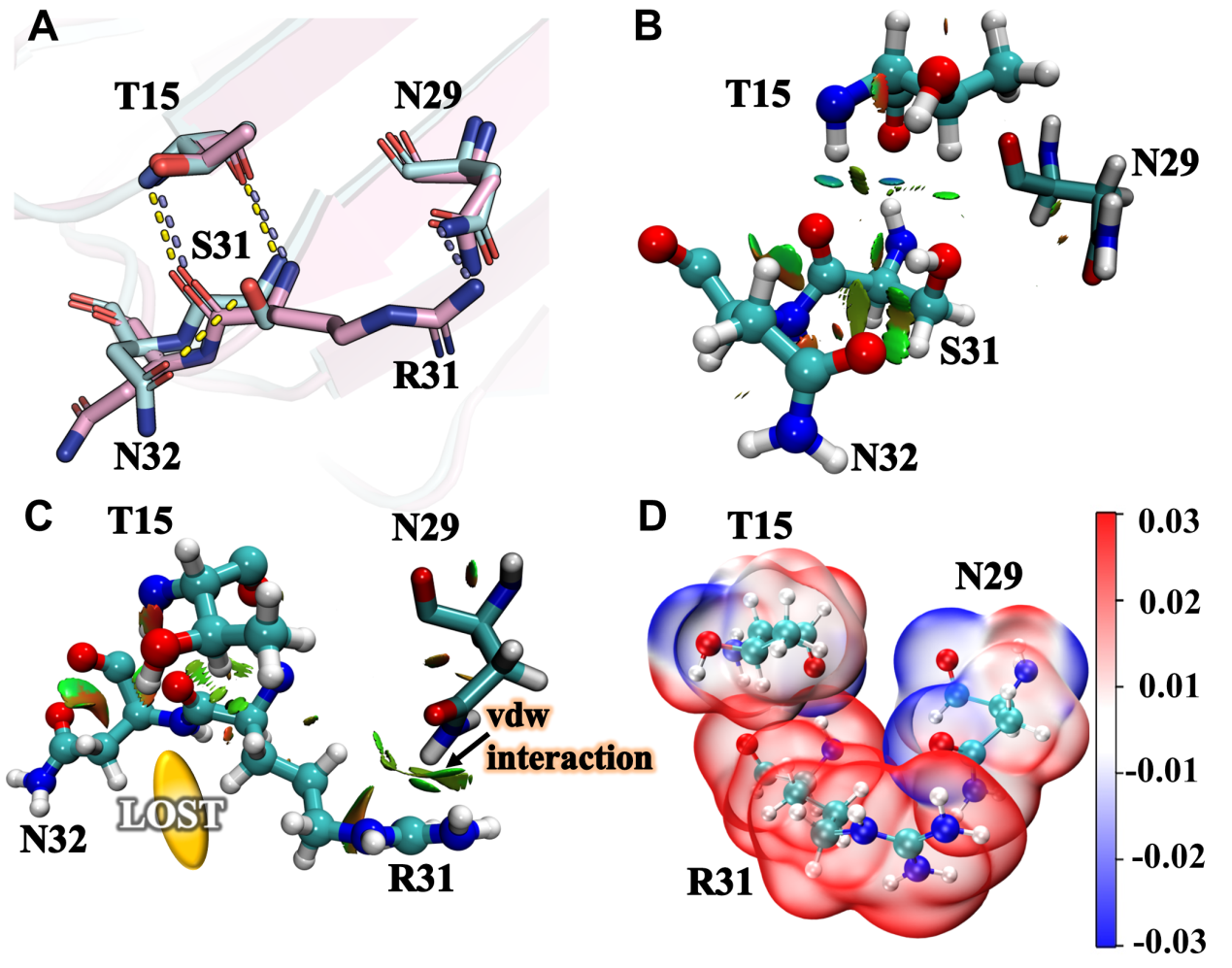
**

**Figure S5**

**
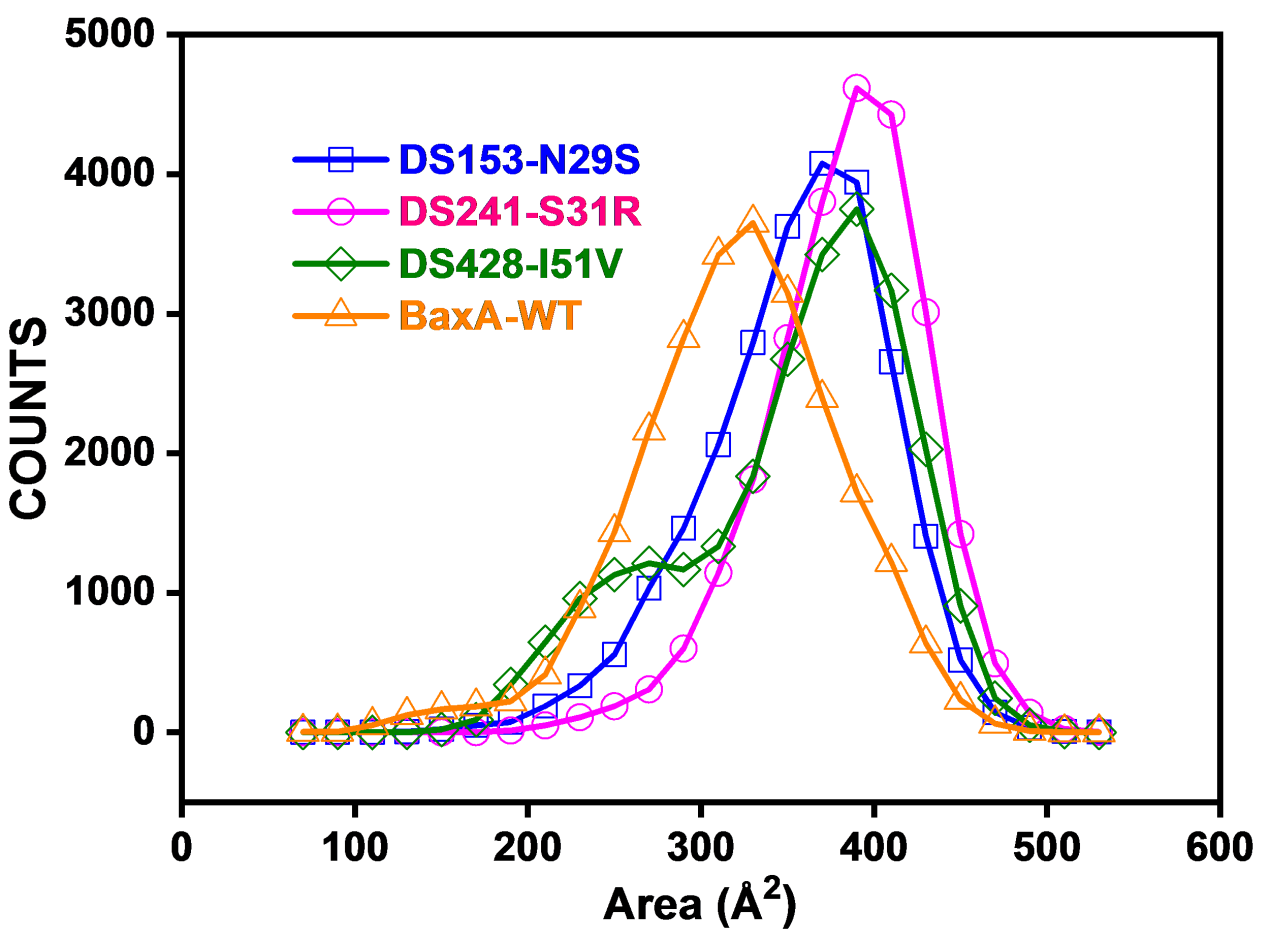
**
